# Transient partial regression of intracranial germ cell tumor in adult thalamus: A case report

**DOI:** 10.3389/fradi.2022.781475

**Published:** 2022-08-31

**Authors:** Si-ping Luo, Han-wen Zhang, Yi Lei, Yu-ning Feng, Juan Yu, Fan Lin

**Affiliations:** Department of Radiology, The First Affiliated Hospital of Shenzhen University, Health Science Center, Shenzhen Second People's Hospital, Shenzhen, China

**Keywords:** germinoma, spontaneous regression, MRI, case report, steroid, immune response

## Abstract

**Background:**

Intracranial germ cell tumors (GCTs) are a relatively rare malignancy in clinical practice. Natural regression of this tumor is also uncommon. We describe a rare case of an intracranial GCT in the thalamus of an adult that showed spontaneous regression and recurrence after steroid therapy.

**Case description:**

A 38-year-old male patient's MRI of the head suggested space-occupying masses in the left thalamus and midbrain. MRI examination revealed demyelination or granulomatous lesions. After high dose steroid treatment, the symptoms improved. The lesions were significantly reduced on repeat MRI, and oral steroid therapy was continued after discharge. The patient's symptoms deteriorated 1 month prior to a re-examination with head MRI, which revealed that the mass within the intracranial space was larger than on the previous image. He revisited the Department of Neurosurgery of our hospital and underwent left thalamic/pontine mass resection on October 16, 2019, and the pathological results showed that the tumor was a GCT.

**Conclusion:**

Intracranial GCTs are rare in the adult thalamus but should be considered in the differential diagnosis. The intracranial GCT regression seen in this case may be a short-lived phenomenon arising from complex immune responses caused by the intervention.

## Introduction

Intracranial germ cell tumor (GCT) is a relatively rare malignancy in the clinic. These tumors are more common in the sellar area and pineal area of adolescents and children, relatively rare in elderly and young children and relatively rare in the thalamus and basal ganglia (1). GCTs can easily cause cerebrospinal fluid diffusion and implant metastasis and invade surrounding structures. These masses are sensitive to radiotherapy but insensitive to steroids (2).

Regarding imaging features, intracranial GCTs usually show equal or long T1 or long T2 signals on MRI. Diffusion-weighted imaging (DWI) typically shows high signal, uniform enhancement, and clear borders, and only a few tumors have moderate or uneven enhancement. GCTs in the basal ganglia have the following unique imaging features: larger tumor, no obvious space-occupying effect, mild peritumoral oedema, and atrophied cerebral cortex of the ipsilateral sylvian fissure. The enhanced scan can show irregular rosette-like enhancement or spot-like enhancement (3). MR spectroscopy (MRS) generally shows a decreased N-acetylaspartate (NAA) peak, increased cholinesterase (Cho) peak, and a towering lipid (Lip) peak.

The incidence of spontaneous regression in tumors is 1/60,000–1/100,000. The central nervous system diseases that require differential diagnosis include primary central nervous system lymphoma, demyelinating pseudotumors, glioma (4), infectious diseases, intracranial parasitic diseases, etc. In addition to intracranial diseases, the natural regression of tumors can occasionally be seen in other parts of the body. According to US statistics, in the 60 years from 1900 to 1960, only ~1,000 cases of spontaneous tumor regression have been reported in the medical literature worldwide. The most common malignant tumors that regress spontaneously are neuroblastoma, choriocarcinoma, kidney cancer and malignant melanoma. These four kinds of malignant tumors account for approximately half of all spontaneous regressions of malignant tumors. The phenomenon of spontaneous tumor regression is rare in intracranial GCTs, and there are only 11 reported cases (5), including our case.

## Case report

### Main complaint

A 38-year-old male patient was admitted to the hospital with weakness in his right limbs for 6 months and decreased vision in his left eye for 3 months.

### History of present illness

The patient developed numbness of the right upper limb without obvious inducement in April 2019 and then experienced continuous numbness without an intermittent period; this was accompanied by weakness of the upper limbs, and then the above symptoms were aggravated with right lower limb weakness, which caused walking instability. In June 2019, the abovementioned symptoms were gradually aggravated with blurred vision and decreased vision in the left eye. In August 2019, the neurosurgery department in our hospital considered the possibility of demyelinating pseudotumors. After high dose dexamethasone treatment, the patient's right limbs remained weak, his visual impairment significantly improved, and the lesion was significantly reduced in size. However, in October 2019, his symptoms worsened, and the lesions appeared larger than before (see Table 1).

**Table 1:** Patient's medical history.

**Time**	**Chief complaint**	**Physical examination**	**MRI expression**	**Laboratory examination**	**Treatment**	**Result**
2019-04	Numbness and weakness of right upper limb, weakness of right lower limb	–	–	–	–	–
2019-06	The numbness and weakness of the right limb with blurred vision and decreased vision in the left eye	–	The left thalamus and midbrain are occupied, diagnosis: malignant tumor	–	–	–
2019-08	Progressive right limb weakness for more than 4 months	Muscle strength of the right limb was grade 4, the deep shallow sensation of the right limb was obviously decreased	Abnormal signal mass showed ring and nodular enhancement. Combined with PWI, DCE, DTI, and MRS, diagnosis: demyelinating pseudotumor or granulomatous lesions	CSF: protein + +, Tumor marker: increased ferritin.	Steroid shock therapy	Symptoms improved
2019-09	The weakness of right limbs was improved	–	The extent of the lesion and the degree of enhancement was reduced. diagnosis: demyelinating pseudotumor or lymphoma	Tumor marker: increased ferritin.	Oral steroid	Symptoms improved
2019-10	The weakness of right limbs and the decrease of left vision were aggravated	The left eyelid drooped, the vision of left eye decreased, the light reflex was slow; right limb is the same as before	The extent of the lesion was larger than that of the former, and the enhancement degree was stronger than that of the former	CSF: protein + +, Tumor marker: increased ferritin	Operation	Pathology: germinoma
Follow up	Now patients in regular chemotherapy, symptoms improved, can live on their own and have a certain ability to work.					

### Past medical history

The patient was healthy and had no history of surgery. His family history was negative, with no congenital diseases.

Physical examination: The following findings were observed: drooping of the left eyelid, decreased visual acuity in the left eye, left pupil:right pupil ratio = 4:2, dull light reflection, level 4 right limb muscle strength, and significantly decreased sensation to both deep and shallow stimuli in the right limb.

Laboratory examination: In August 2019, routine blood tests, electrolytes, liver and kidney function, coagulation function, tumor markers, and electrocardiography showed no obvious abnormalities. The cerebrospinal fluid analysis revealed significantly increased levels of trace total protein, but there were no obvious abnormalities. Ferritin was significantly increased, but the CA125, CA199, CA153, CEA, and AFP levels were not abnormal.

### Imaging examination

In June 2019, a head MRI performed at an external hospital showed a lesion occupying the left thalamus and midbrain space. Considering the possibility of malignant tumors, PET-CT was performed and showed that tumor-like changes with demyelination were likely.

**Figure 1:**
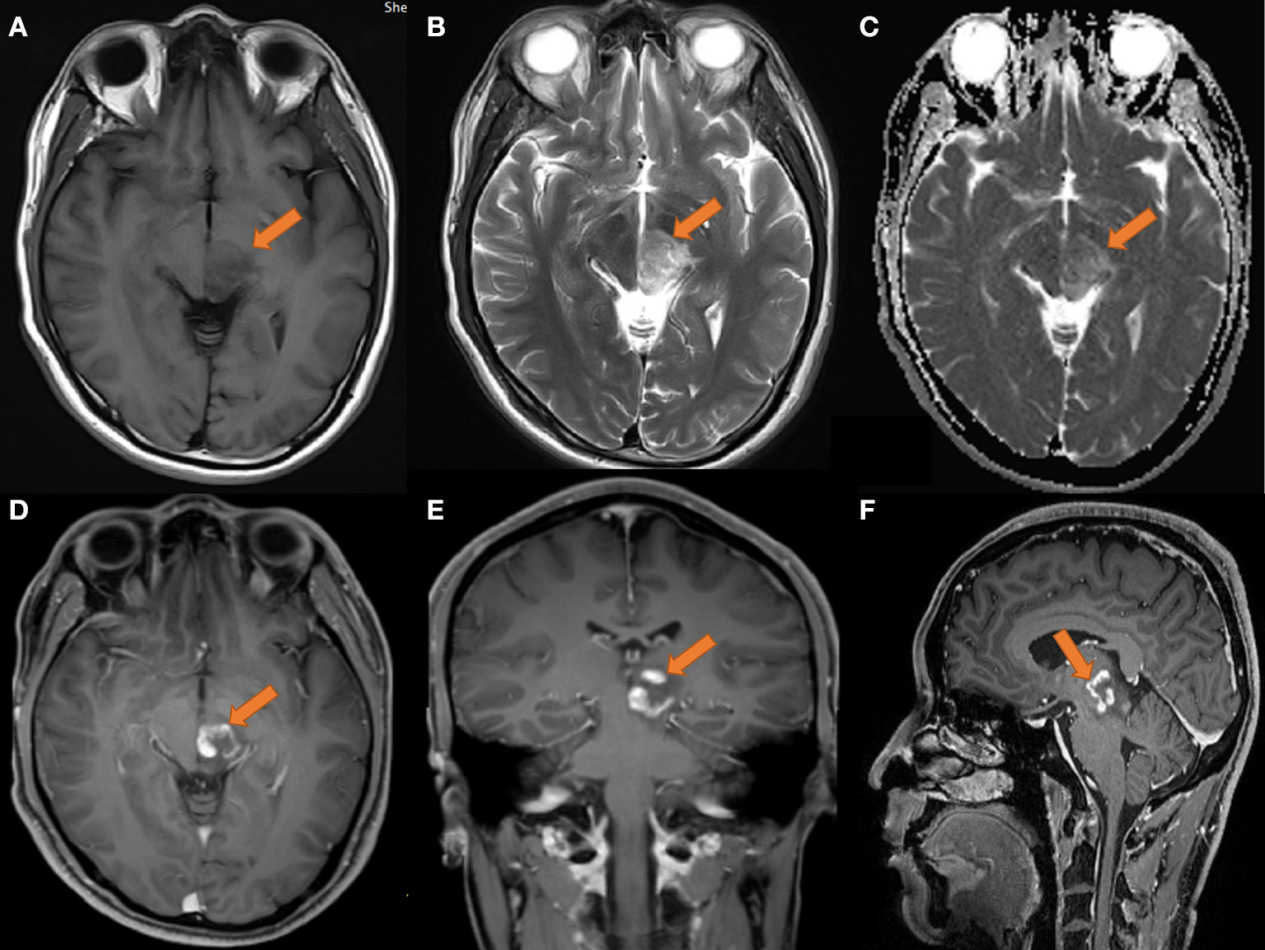
MRI of the head on 2019-08-14. **(A)** T1 transverse view: patchy low signal mass with unclear margin in left thalamus and pons. **(B)** T2 transverse section: the lesions showed moderate and slightly high signal. **(C)** ADC showed limited diffusion; **(D–F)** transverse coronal sagittal enhancement: the lesions showed ring-shaped enhancement, with open ring sign; no obvious necrosis was found in the left thalamus and pons; the size of the lesion was about 25 × 23 × 21 mm.

The MRI in August 2019 (as shown in Figure 1) revealed that the left thalamus and pontine had medium-to-low T1WI signals and medium-to-slightly high T2WI signals, and the enhancement appeared ring-like, nodular and irregular. Perfusion-weighted imaging (PWI) showed that the lesion area had hypoperfusion, the cerebral blood volume (CBV) and cerebral blood flow (CBF) were not significantly increased, and the mean transit time (MTT) and time-to-bolus peak (TTP) were delayed. On MRS, areas of interest were selected from the lesion and the contralateral area with normal contrast. The spectrum line shows that the spectrum in the left lesion was abnormal, the Cho peak was higher in the lesion than in the contralateral normal area, the NAA peak was significantly lower than that in the corresponding area, and the Lip peak was visible. Based on imaging, demyelinating pseudotumors or granulomatous lesions should be considered.

**Figure 2:**
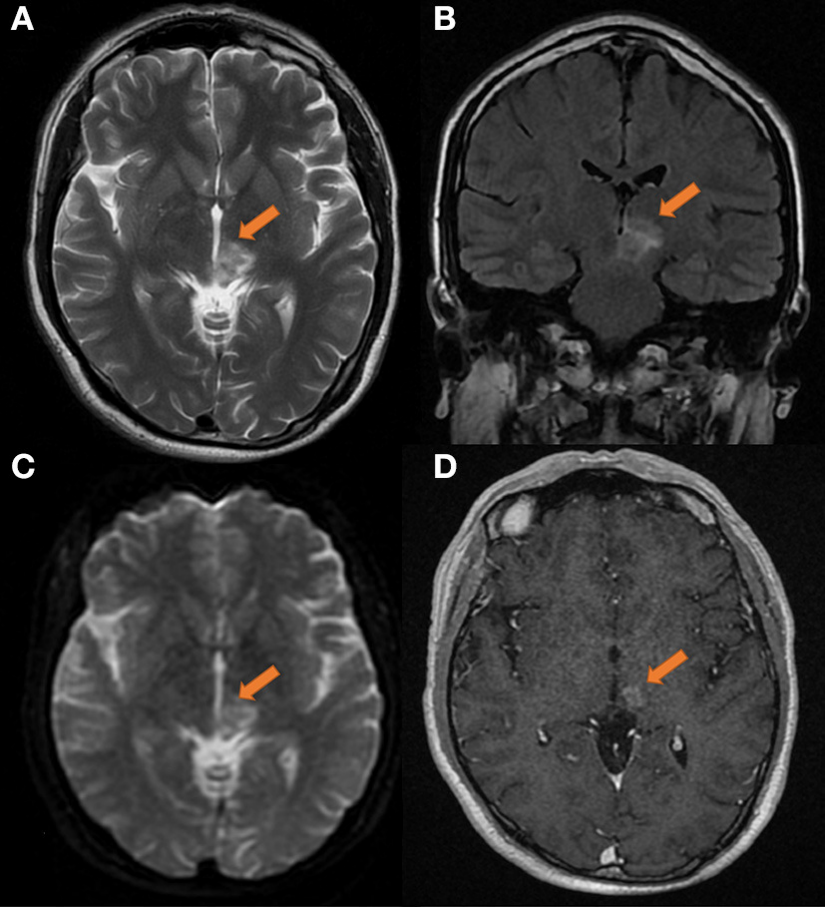
Head MRI on 2019-09-03, compared with the anterior film (2019-08-14). **(A)** T2 transverse view: patchy medium and high signal masses can be seen in the left thalamus and pons, with smaller range and unclear edge. **(B)** T2 FLAAR lesions showed moderate and slightly high signal intensity. **(C)** DWI showed high signal intensity. **(D)** Transverse enhanced: the degree of enhancement of the lesions was less than before, showing nodular appearance, and no obvious necrosis was found in the lesions. The size of lesions was about 20 × 16 × 20 mm.

Surprisingly, a re-examination of the cranial MR findings in September 2019 (as shown in Figure 2) revealed that the extent of the left thalamic and pontine lesions was reduced compared to that on the initial images, and the margins were unclear. After applying contrast enhancement, the degree of enhancement was lower than that of the previous scan, and nodules were observed. Similarly, no obvious necrosis was seen inside. Considering that the possibility of demyelinating pseudotumors was high, lymphoma could not be completely excluded.

**Figure 3:**
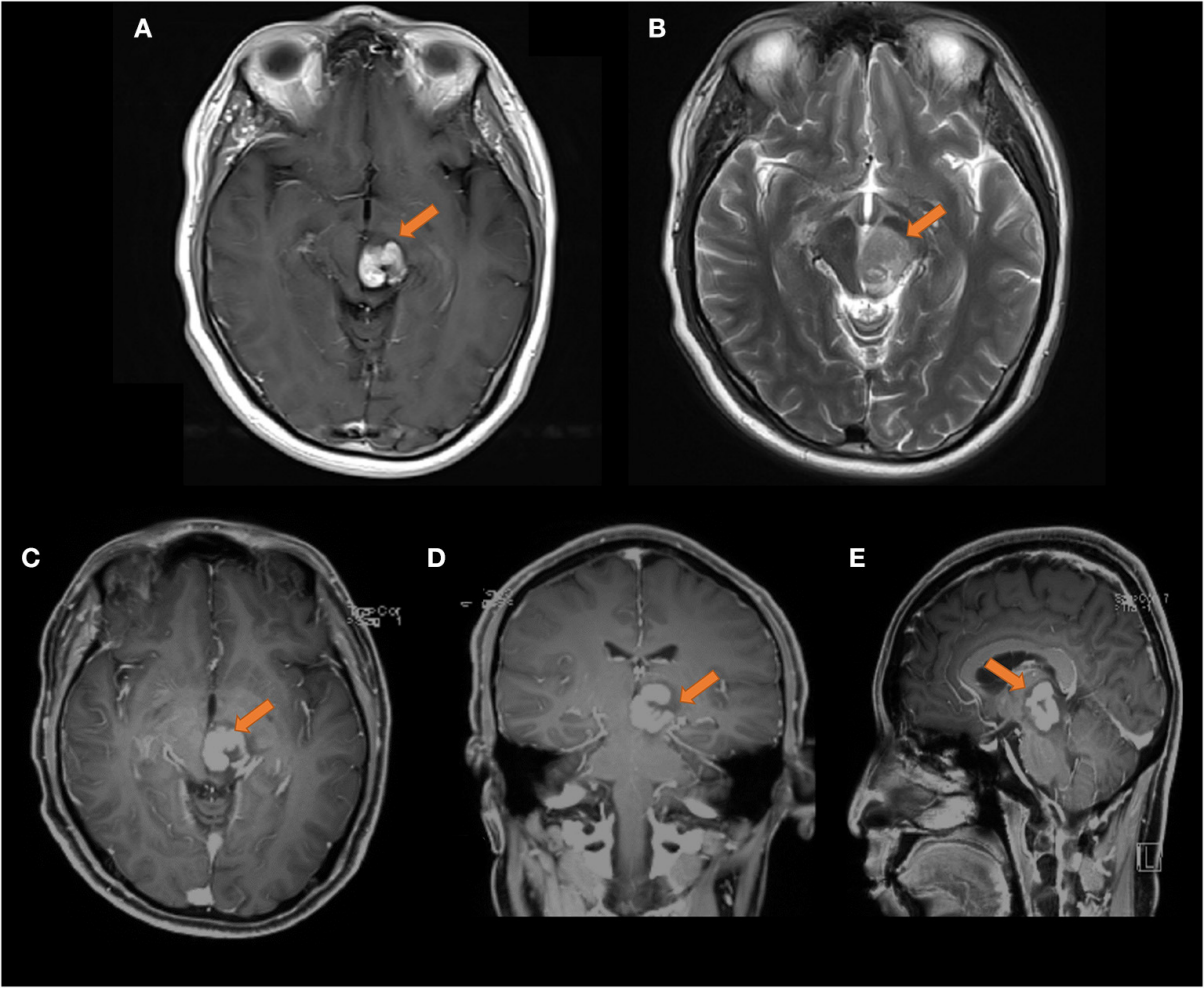
Head MRI on 2019-10-08, compared with the front film (2019-09-03). **(A)** T1 transverse view: patchy medium high signal mass can be seen in the left thalamus and pons, the range is larger than the front, and the edge is not clear. **(B)** T2 transverse section: the lesions show moderate and slightly high signal. **(C–E)** Transverse coronal sagittal enhancement: the degree of enhancement of the lesion is higher than that of the previous film, and the lesion area is larger than the previous film, with obvious space occupying effect The size of the lesion was about 23 × 21 × 30 mm.

However, the cranial MR findings from October 2019 (as shown in Figure 3) showed that the left thalamus and pontine lesions were enlarged compared to those on the previous scan, and the edges were unclear. After the application of contrast enhancement, the degree of enhancement was stronger than before, and a nodular pattern was observed. There were obviously necrotic foci on the image, which typically suggest lymphoma.

### Treatment

**Figure 4:**
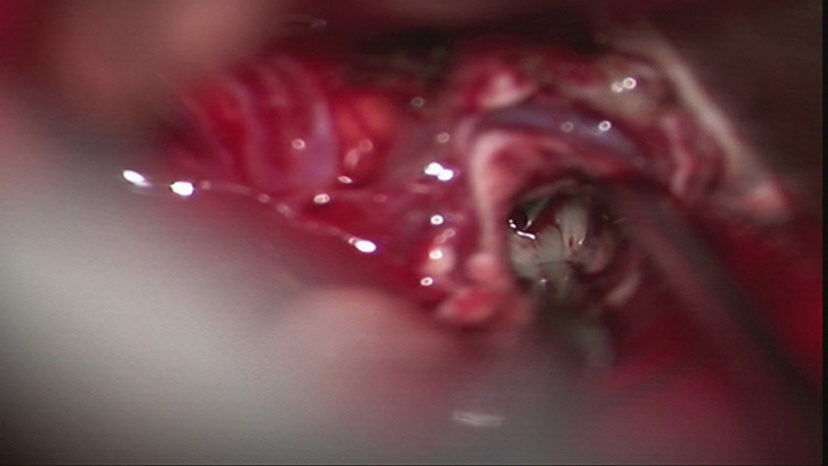
During the operation on October 16, 2019, gray white lesions were found.

In October 2019, under general anesthesia, the patient underwent total tumor resection, resection of left thalamus and pons lesions (Figure 4). Hemostasis was achieved, and preventative treatment for epilepsy was given after the operation.

**Figure 5:**
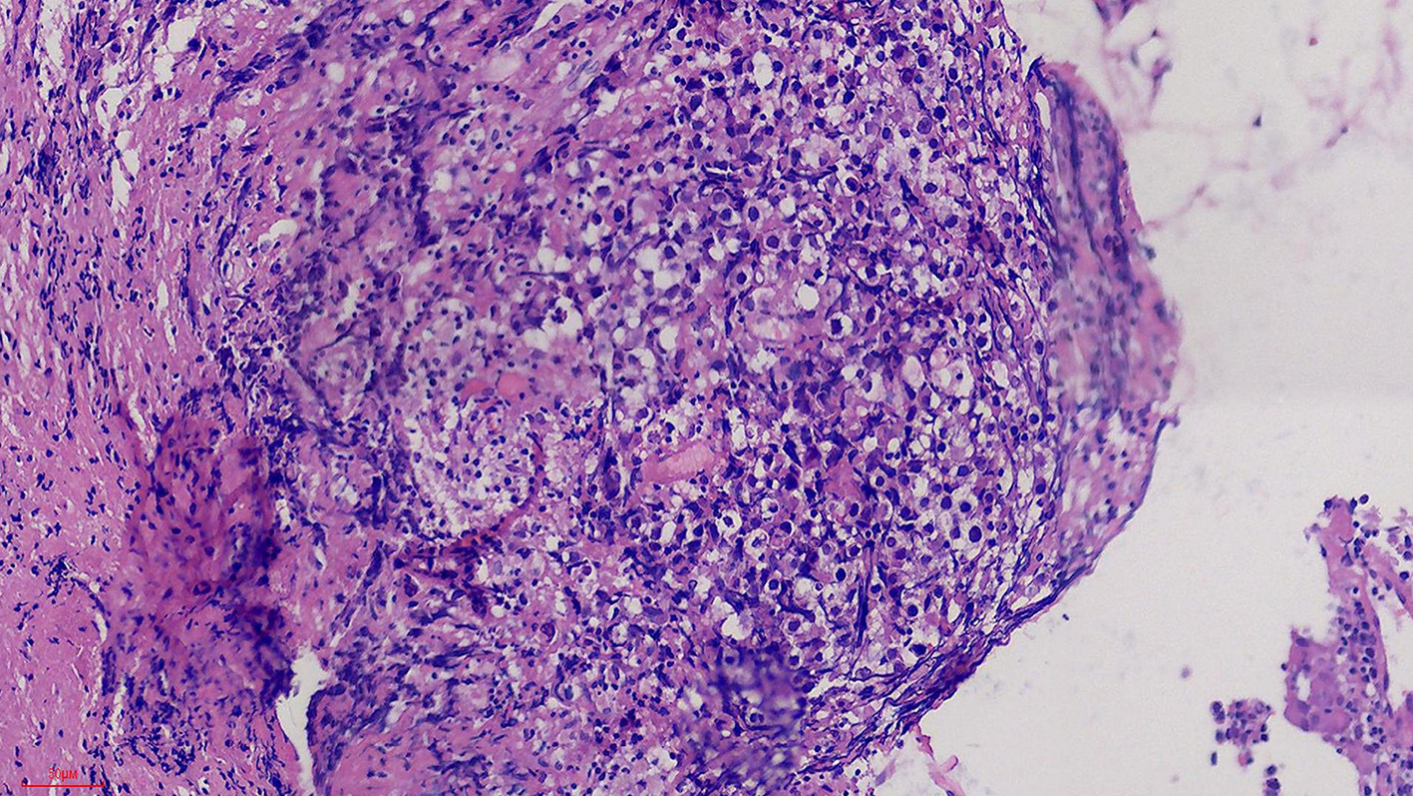
Histological sections. 20 × Hematoxylin and eosin stained sections, which showed that the tumor cells were diffusely proliferated, with large, round, or oval nuclei, obvious nucleoli, visible mitosis, and more lymphocytes infiltrating in the stroma.

Results and follow-up: The postoperative pathological analysis (as shown in Figure 5) revealed that the tumor cells were diffusely proliferated, had large nuclei, were round or oval, and had obvious nucleoli, visible mitosis, and more lymphocyte infiltration in the interstitium. The immunohistochemistry results were as follows: GFAP(-), Ki67 (~60%+), LCA(-), PLAP(+), OCT3/4(+), and SALL4(+); these findings were consistent with those of a GCT. The patient is now undergoing regular chemotherapy, with improvements in his symptoms, and he can live on his own and has the ability to work. There was no definite recurrence in postoperative follow-up.

## Discussion

This case was not diagnosed as germinoma before operation. The reasons for the misdiagnosis in this case (as shown in Table 2) are as follows: ① the incidence of GCTs is low, accounting for ~1% of intracranial tumors. GCTs mainly occur in young people, and 90% occur in individuals under 20 years old (1). The patient in this case was 38 years old, so the age of onset was atypical. The clinical symptoms of the patient significantly improved, and the lesions significantly shrank after high dose steroid therapy. Combined with PET-CT, the examinations suggested the possibility of demyelinating pseudotumors. ② One month after steroid therapy, the patient's clinical symptoms were aggravated, and the lesions were significantly enlarged. Based on the imaging features and response to lymphoma steroid therapy, the diagnosis was likely to be lymphoma, not GCTs. ③ GCTs mostly occur in the pineal area and the saddle, and the focus in this case occurred in the thalamus and pons; thus, the onset location was atypical. ④ GCTs are prone to cerebrospinal fluid seeding and dissemination. In this case, no tumor cells were detected by cerebrospinal fluid cytology.

**Table 2:** Comparison of the characteristics of germinoma and this case.

	**Germ cell tumor**	**This case**	**Match or not**
Onset age	Children and adolescents, common in 10–25 years old more	38 years old	No
Predilection site	Pineal, suprasellar, or basal ganglia regions	Left thalamus and pons	No
Metastasis pathway	Invasive growth, easy CSF dissemination	No tumor cells were found in CSF	No
First symptoms	Intracranial hypertension, diabetes insipidus, hemiplegia, or hemiparesthesia	Weakness of right limb	Yes
CT features	Round mass with equal or slightly high density, accompanied by calcification and multiple cysts	–	–
MRI features	T1WI (equal or low signal) T2WI (high signal) DWI (equal or slightly high signal) Homogeneous enhancement.	T1WI (low signal) T2WI (high signal)	No
MRS features	NAA decreased, Cho increased, high Lip appeared	Cho increased, NAA decreased significantly	Yes
Treatment	Sensitive to radiotherapy but not to steroid	After steroid treatment, the lesion was significantly reduced	No

Gliomas and lymphoma are common tumors in basal ganglia. The imaging features are consistent with this case, and the lesions can be reduced after steroid therapy. CSF, cerebrospinal fluid; NAA, N-acetylaspartic acid; Cho, cholinesterase; Lip, lipid.

The common differential diagnoses are shown in Table 3. Among the possibilities for this case, lymphoma was the most likely. Lymphoma usually starts with neurological dysfunction, and the MRI manifestation typically includes low T1 signals and high T2 signals. After applying contrast enhancement, obvious, and uniform enhancement could be seen. The edge of the enhancement was not as sharp and regular as that of meningioma. When the enhancement was not obvious, it indicated tumor necrosis. DWI showed a uniform high signal, PWI showed low perfusion, and MRS showed a Lip peak. Moreover, lymphoma is sensitive to steroid therapy. In contrast to the diagnosis based on the significant response of demyelination-type diseases (6) to steroid therapy, the imaging examinations performed after the clinical use of steroid therapy in this case revealed the recurrent phenomenon of lesion reduction, enhancement and reduction of the lesion, and subsequent enlargement of the lesion, which was consistent with the characteristics of lymphoma. Therefore, these findings led to a misdiagnosis in this case.

**Table 3:** Common differential diagnosis as follow.

**Lesions**	**MRI features**						**Predilection sites**	**Pathology**
	**T1WI**	**T2WI**	**CE**	**DWI**	**PWI**	**MRS**		**Immunohistochemical**
Glioma	Hypo	Hyper	Mild, marked	Mild‵ Obviously limited	HP	NAA↓↓ Cho↑↑	Whole brain	IDH1 (+) TERT (+) GFAP (+) MGMT (+)
Lymphoma	Iso‵ Hypo	Iso‵ Hyper	Marked	Obviously limited	LP	Lip↑↑	Midline, basal ganglia, paraventricular	LCA (+) CD45 (+) CD11A/18 (+)
Demyelination	Iso‵ Hypo	Iso‵ Hyper	Mild, moderate, and marked	Peripheral limited	LP	Cho↑	Whole brain	–
Intracranial germinoma	Iso‵ Hypo	Iso‵ Hyper	Homo, hetero, Rosette like, or spotted enhancement (basal ganglia region)	Obviously limited	LP	NAA↓↓ Cho↑↑ Lip↑↑	Pineal region, suprasellar region	PLAP (+) OCT3/4 (+) SALL4 (+) LCA (–)

CE, contrast enhancement; HIS, high intensity signal; Homo, homogeneous; Hetero, heterogeneous; Hyper, hyperintense; Hypo, hypointense; Iso, isointense; HP, high perfusion; LP, low perfusion; NAA, N-acetylaspartic acid; Cho, cholinesterase; Lip, lipid.

The phenomenon of tumor reduction seen in this case is known as “spontaneous regression” in medicine. This is a rare phenomenon. The tumor regression of intracranial GCTs may be due to their good response to radiotherapy, which is reflected by the imaging manifestation of tumor volume reduction. It has been speculated in the literature (7) that so-called spontaneous resolution may be the iatrogenic transient consequence of medical interventions such as radiological examinations, surgery, steroids, and immune responses.

In this regard, we make the following inferences.

First, for simple GCTs with high radiosensitivity, a single cycle of radiotherapy (11 Gy or 10 Gy, 5 times) can significantly shrink the tumor (8). In similar cases, diagnostic radiation, including repeated CT scans and angiography, was administered before tumor regression. However, the radiation dose of a single CT scan is very low, usually considered to be <0.05 Gy (5). Although the patient in this case underwent a PET-CT examination, we believe that such a low dose of radiation is not enough to cause tumor regression. Furthermore, some authors (9) have reported that some patients undergo surgery before the tumor subsides, such as ventricular-abdominal shunt placement, lateral ventricle puncture and external drainage, or tumor biopsy. They believe that surgery can affect the patient's immune status, leading to local inflammatory cell accumulation and antitumor effects such as lymphocyte infiltration, which may cause an inflammatory antitumor response and lead to tumor regression. However, there have been 4 cases of tumor regression without surgery, in addition to our case. Therefore, surgical intervention is not the reason for spontaneous regression.

Second, steroids are usually ineffective for GCTs. In our case, the mass was considered a demyelinating pseudotumor and lymphoma before operation. Because of the use of high dose steroid therapy, the patient's symptoms improved, and the phenomenon appeared resolved on MRI. For demyelinating pseudotumors (6), lymphoma and the GCT in this case, the clinical use of high dose steroid therapy may produce different results. Steroid therapy is generally effective for demyelinating pseudotumors. After steroid treatment, the symptoms improved, the lesions shrank, and there was generally no lesion recurrence. Lymphoma (7) is usually sensitive to steroids. After steroid treatment, the symptoms will improve first, the lesions will shrink, and then the lesions will increase in size again. Masoudi et al. (10) reported that these tumors mostly resolve 14 days after the use of steroids. The reason for the effect in this case was not from the effect of the steroids on the cells of the lesion itself but the cytotoxic effect on the lymphocytes infiltrating around the tumor. In this case, pathological light microscopy showed that there were many lymphocytes infiltrating the stroma of the tumor. Steroid treatment may cause an inflammatory antitumor response, leading to temporary regression of the lesion, as seen on MRI. A previous case (11) is similar to our report. In patients with GCT, the lesion will shrink after steroid treatment, and then the lesion will increase again. However, similar to the analysis of the effects of the aforementioned surgical approaches, four patients in the literature did not receive steroid therapy and experienced spontaneous regression, so the role of steroids in natural regression needs further study.

Finally, the complex immune response of the above factors with human immune responses and tumors may be the reason for natural regression (12). However, there is no direct evidence to prove this. In future research, maybe we can do research on this aspect.

## Conclusion

Intracranial GCTs occur in the adult hypothalamus, and regression is extremely rare. This case of GCT was misdiagnosed because of an atypical appearance on MRI and “regression” and recurrence after steroid therapy. We speculate that the regression observed may be an iatrogenic transient phenomenon arising from the complex immune response caused by the intervention. Therefore, it is necessary to be aware of the possible impact of iatrogenic intervention and that it may cause regression of intracranial GCTs, especially when patients show improved symptoms. Before providing further treatment, more imaging tests should be performed to reduce the incidence of misdiagnosis.

## Learning points

Intracranial GCTs occur in the adult hypothalamus, and regression is extremely rare. This case of GCT was misdiagnosed because of an atypical appearance on MRI and “regression” and recurrence after steroid therapy.

## Data availability statement

The original contributions presented in the study are included in the article/supplementary material, further inquiries can be directed to the corresponding authors.

## Ethics statement

The studies involving human participants were reviewed and approved by the Clinical Research Ethics Committee of Shenzhen Second People's Hospital. The patients/participants provided their written informed consent to participate in this case study and for the publication of this case report.

## Author contributions

S-pL contributed to the conception of the study. Y-nF, H-wZ, and YL contributed significantly to the analysis and manuscript preparation. FL performed the data analyses and wrote the manuscript. JY helped perform the analysis with constructive discussions. All authors contributed to the article and approved the submitted version.

## Funding

This study was supported by a grant from the Basic Plan Program of Shenzhen, China (No. JCYJ20180228163333734) and Clinical Research Project of Shenzhen Second People's Hospital, China (No. 20193357021).

## Conflict of interest

The authors declare that the research was conducted in the absence of any commercial or financial relationships that could be construed as a potential conflict of interest.

## Publisher's note

All claims expressed in this article are solely those of the authors and do not necessarily represent those of their affiliated organizations, or those of the publisher, the editors and the reviewers. Any product that may be evaluated in this article, or claim that may be made by its manufacturer, is not guaranteed or endorsed by the publisher.
